# Recruitment of a critically endangered sawfish into a riverine nursery depends on natural flow regimes

**DOI:** 10.1038/s41598-019-53511-9

**Published:** 2019-11-19

**Authors:** Karissa O. Lear, Adrian C. Gleiss, Jeff M. Whitty, Travis Fazeldean, J. R. Albert, Nathan Green, Brendan C. Ebner, Dean C. Thorburn, Stephen J. Beatty, David L. Morgan

**Affiliations:** 1 Centre for Sustainable Aquatic Ecosystems, Harry Butler Institute, Murdoch University, 90 South Street, Murdoch, WA 6150 Australia; 2 Environmental and Conservation Sciences, Murdoch University, 90 South Street, Murdoch, WA 6150 Australia; 3 Nyikina-Mangala Rangers, Walalakoo Aboriginal Corporation, PO Box 1115, Derby, WA 6728 Australia; 4 Centre for Tropical Water and Aquatic Ecosystems Research, James Cook University, 1 James Cook Drive, Douglas, QLD 4814 Australia; 5 Indo-Pacific Environmental, 1/194 Scarborough Beach Road, Mount Hawthorn, WA 6016 Australia

**Keywords:** Behavioural methods, Conservation biology, Hydrology

## Abstract

The freshwater sawfish (*Pristis pristis*) was recently listed as the most Evolutionarily Distinct and Globally Endangered (EDGE) animal. The Fitzroy River in the remote Kimberley region of north-western Australia represents a significant stronghold for the species, which uses the freshwater reaches of the river as a nursery. There is also mounting pressure to develop the water resources of the region for agriculture that may substantially affect life history dynamics of sawfish in this system. However, the relationship between hydrology and population dynamics of freshwater sawfish was unknown. We used standardized catch data collected over 17 years to determine how wet season volume influences recruitment of freshwater sawfish into their riverine nursery. Negligible recruitment occurred in years with few days of high flood levels (above 98^th^ percentile of cease-to-flow stage height), and relatively high recruitment occurred in years with 14 or more days of high flood levels. This relationship is indicative of a distinct boom-or-bust cycle, whereby freshwater sawfish rely almost entirely on the few years with large wet season floods, and the brief periods of highest water levels within these years, to replenish juvenile populations in the Fitzroy River nursery. This has direct implications for sustainable water resource management for the Fitzroy River basin in order to preserve one of the last known intact nursery habitats for this globally threatened species.

## Introduction

Freshwater environments are among the most threatened ecosystems worldwide, with greater biodiversity loss documented in these habitats than in the most affected marine or terrestrial ecosystems^1–4^. Although this loss of biodiversity can be attributed to a number of anthropogenic factors, for many freshwater ecosystems land use poses the greatest threat^1,5,6^. This is particularly true for arid regions, where human needs for water are at their highest and natural flow regimes are often the most variable^6–8^. As a result, dryland river systems are among the most threatened of all freshwater ecosystems^9^.

Dryland rivers are characterized by low annual rainfall and highly dynamic, sometimes unpredictable, flow regimes, which present inherent difficulties to humans in harnessing reliable water resources from these systems. Water resource developments, including dams, water extraction, or flow diversion, mediate this natural variability to create useable resources for human purposes. However, these structures and processes may substantially change the flow dynamics and hydrological characteristics of the systems^1,10,11^, which can have enormous consequences for aquatic species whose ecologies and life histories are often intricately connected to variable flow regimes^12–14^.

Owing to the dynamic nature of dryland river systems, understanding hydro-ecological relationships within these ecosystems is challenging^9^. Such dynamic environments show substantial inter-annual variation in environmental conditions and resource availability, and typically host species that have evolved strategies to deal with the dynamics of the water and energy supply regime^14,15^. Determining how these species interact with their ecosystem and cope with the inherent extreme environmental variation therefore requires ecological studies spanning long time periods in order to reveal the full extent of important ecological processes and population dynamics^16,17^. While such long-term monitoring of environmental conditions such as rainfall, river discharge rates, and temperature are relatively common, there is a dearth of long-term ecological data, which makes it difficult to link the environmental variability to ecological processes.

Among the long-lived, dryland river species is the freshwater sawfish, *Pristis pristis*. This circumtropically distributed diadromous species has undergone a range retraction of >60%, largely driven by overexploitation and habitat loss^18^, leading to its listing as Critically Endangered by the International Union for the Conservation of Nature (IUCN)^19^, and as the most at risk species on the Evolutionarily Distinct and Globally Endangered (EDGE) list (Zoological Society of London; https://www.edgeofexistence.org/). The freshwater sawfish is the only sawfish species that inhabits freshwater environments during part of their lifecycle, using riverine habitats as nurseries^20,21^. Remote parts of northern Australia offer some of the last known viable populations of this species within its remaining range^20,22,23^. The Fitzroy River in the remote and sparsely populated Kimberley region of Western Australia has been identified as a particularly important nursery habitat for the species; with substantially more individuals documented in this river system than any other in northern Australia or elsewhere in the world^20,22–24^. Importantly, this species also holds substantial cultural and spiritual significance to the traditional owners of the Kimberley, such as the Bunuba, Gooniyandi, Nyikina, and Walmajarri people in the Fitzroy valley^21,22,25^, as well as human interest across a number of other audiences and perspectives. For example, the freshwater sawfish has been identified as a promising ‘flagship species,’ that could be harnessed to enhance awareness and engender conservation efforts in freshwater environments^26^.

The Fitzroy River is a dryland river that operates under a monsoonal climate with extreme wet and dry seasons. Like many dryland rivers, it also has substantial inter-annual variability in discharge^13,14,27^, with annual wet season discharge varying more than 25 fold over the last 20 years (Government of Western Australia River Monitoring Stations; http://kumina.water.wa.gov.au). Such variability in discharge rates has been shown to affect recruitment and abundance of a variety of dryland river fish taxa^9,10,28,29^, although the relationship between river discharge and sawfish abundance has not been previously examined. Due to the low human population (<20,000 people across 100,000 km^2^), the Fitzroy River catchment and its estuary have historically had a low level of anthropogenic disturbance compared to other rivers globally, including in Australia, and the river system is largely unregulated, which is likely to be a major factor contributing to the relatively high local sawfish abundance^20^. However, this river system has recently been the subject of numerous water extraction proposals related to agriculture and mining developments, and therefore, there is a sense of urgency in determining the relationship between river flow rates and sawfish population dynamics. Moreover, the freshwater sawfish is listed nationally as Vulnerable under the Environment Protection and Biodiversity Conservation Act 1999, thus it is crucial to determine how this species interacts with the dynamic hydrological conditions in the river in order to understand the ecological implications of these proposals.

The present study uses long-term catch data from standardized freshwater sawfish surveys in the Fitzroy River to determine how river flow dynamics affect recruitment of freshwater sawfish into their riverine nurseries. Determining the relationship between hydrological parameters associated with wet season flood volumes and recruitment rates will help to inform the sustainable development and water allocation of the Fitzroy River, globally one of the last known intact nursery habitats for this highly threatened species.

## Materials and Methods

All work with animals was conducted under permits granted by Western Australia Department of Fisheries and Murdoch University’s Ethics Board and all work adhered to the relevant guidelines and regulations.

### Study site

The Fitzroy River in Western Australia is a highly dynamic system. During the wet season, spanning approximately December to May, monsoonal rains fill the catchment and the river is fully connected. However, during the dry season (approximately June to November) river flow decreases and eventually ceases, forming distinct permanent pools that are fed only by local aquifers^30^. Freshwater sawfish have been observed in these dry season pools up to 450 km from the river mouth^22^. The study sites were located in the lower 400 km of the Fitzroy River, noting that the lower 17 km is tidally influenced. Further upstream water is fresh (<1 ppt).

Freshwater sawfish are pupped in the macrotidal estuary of King Sound during the wet season, and migrate into the Fitzroy River estuary and then into the non-tidal, freshwater reaches of the river^20,21^. They use the freshwater reaches as a nursery for 4–6 years, reaching between 2.2 and 2.6 m total length before returning to marine environments to mature^20,21,31,32^.

### Sawfish surveys

**Figure 1:**
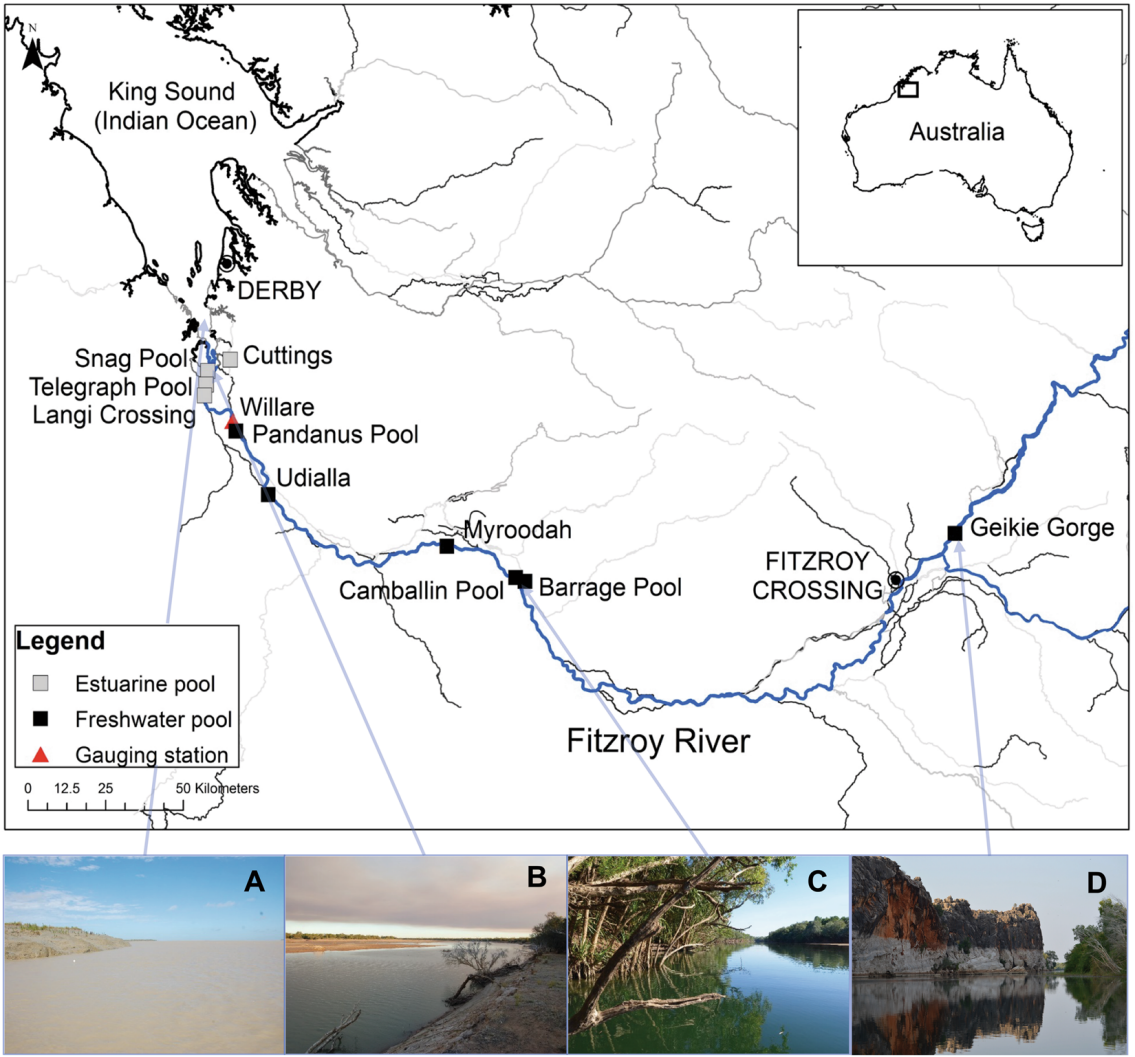
Map (ArcGIS 10.2.1; https://esriaustralia.com.au/arcgis-desktop) of sawfish survey locations in the Fitzroy River. Estuarine and freshwater survey locations are shown, along with the hydrological data collection site (Willare; red marker). Photographs show examples of habitat found in (**A**) King Sound near the river mouth, (**B**) Snag Pool (estuarine), (**C**) Camballin Pool (freshwater), and (**D**) Geikie Gorge (freshwater) (Photographs: D. Morgan).

Sawfish catch data were collated from targeted surveys conducted over 17 years between 2002–2018 in the Fitzroy River. Sawfish surveys occurred between April and December, but most commonly in June/July (early dry season) and October/November (late dry season). Surveys were conducted in estuarine sites including Snag Pool, Telegraph Pool, Langi Crossing, and Cuttings, and freshwater pools including Udialla, below Myroodah Crossing, below the Camballin Barrage, Pandanus Pool, Camballin Pool, and Geikie Gorge (Fig. 1). Survey methods consisted of the use of 20-m panels of 51 to 203 mm stretched monofilament gill nets, but in most years 156 mm mesh was used. Nets were often adjoined to create a total net length of 40 to 80 m, and reached to the river bed at all sites. Soak time of nets varied, but typically ranged between 1 to 2 hours. Nets were monitored and checked if activity was observed in the net (i.e. a fish was caught). Baited hook and line methods were also used in the surveys as were cast nets on a few occasions when sawfish were observed in shallow water.

Upon capture, sawfish were secured at the caudal fin by hand and at either the base of the rostrum by hand or at the tip of the rostrum by use of an entangled gill net (prior to the removal of the net from the rostrum). The spiracles, mouth and gills remained submerged during the handling of sawfish to allow for continued respiration. Morphometric data including total length (TL) and sex were recorded. Rototags (Dalton Tags, Nottinghamshire, UK) were attached to the first dorsal fins of animals^32^, and were individually numbered to assess recaptures of individuals within and between years. Once measured and tagged, sawfish were released at the site of capture.

### Catch per unit effort

In order to compare recruitment rates of freshwater sawfish during the survey period, catch per unit effort (CPUE) of young-of-the-year (YOY) freshwater sawfish in each year was calculated. YOY sawfish were defined as individuals measuring <1300 mm TL^20,21^. CPUE was calculated as the total number of YOY freshwater sawfish caught compared to the total number of net hours fished, as # sawfish 20-m net^−1^ h^−1^. CPUE was derived separately for the early dry season (April–August) and late dry season (September–December) in each year. CPUE was also calculated separately for freshwater and estuarine pools in each year. Additionally, there were differences in survey effort and timing from year to year. Therefore, to standardize data throughout the study period, only the first two days of fishing in any one location on a given trip were included in CPUE calculation.

### Wet season hydrological characteristics

Water level and discharge data for the Fitzroy River were collected from the Department of Water and Environmental Regulation river monitoring stations (kumina.water.wa.gov.au/waterinformation/telem), which record daily discharge and stage height data from telemetered stations in Western Australia. Data were extracted from Willare (station 802008) in the lower catchment (see Fig. 1). Total wet season river discharge was calculated for each year of the study as the sum of river discharge observed between December and May in each year.

### Statistical analyses

Statistical modelling was conducted in R (R Core Team, 2018), using the ‘nlme’^33^ and ‘segmented’^34^ packages. To determine the shape of the relationship between recruitment and river discharge, linear, exponential, power, and segmented linear models were each fit to the data, using the YOY CPUE for freshwater pools in the early dry season only to specifically assess recruitment rather than affects from a combination of recruitment and survival. Total wet season discharge was used as the initial explanatory variable in these models based on past studies which have determined the importance of river discharge for the recruitment of other fish taxa^9,10,28^. The fit of these models was compared using the corrected Aikake’s information criterion (AICc), log likelihood, and R^2^ of each model.

Once the shape of this relationship was determined, the best fit model was used to assess which qualities of the wet season were most informative for predicting recruitment. In this set of models, CPUE was predicted by different variables describing the wet season, including total wet season discharge, discharge during the early (December-January), mid (February-March), and late wet season (April-May), peak stage height, and the number of days during the wet season that the river exceeded the 50^th^, 75^th^, 90^th^, 95^th^ and 98^th^ percentiles of river stage height on record at Willare (1998–2018). Each wet season variable was fit as a separate model, as all variables showed significant collinearity with each other (variance inflation factors >30). Models were compared using AICc, log likelihood, and R^2^.

Additionally, length-frequency histograms of sawfish were created for each year of the study. These histograms included all live sawfish caught during each year, including gill net, handline, and cast net captures. However, individual sawfish caught multiple times within a year (i.e. recaptured fish, determined by Rototag identification) were only included once per year. These histograms were examined to determine whether the dominant size class found in the Fitzroy River in a given year was related to years with high calculated recruitment rates.

## Results

**Figure 2:**
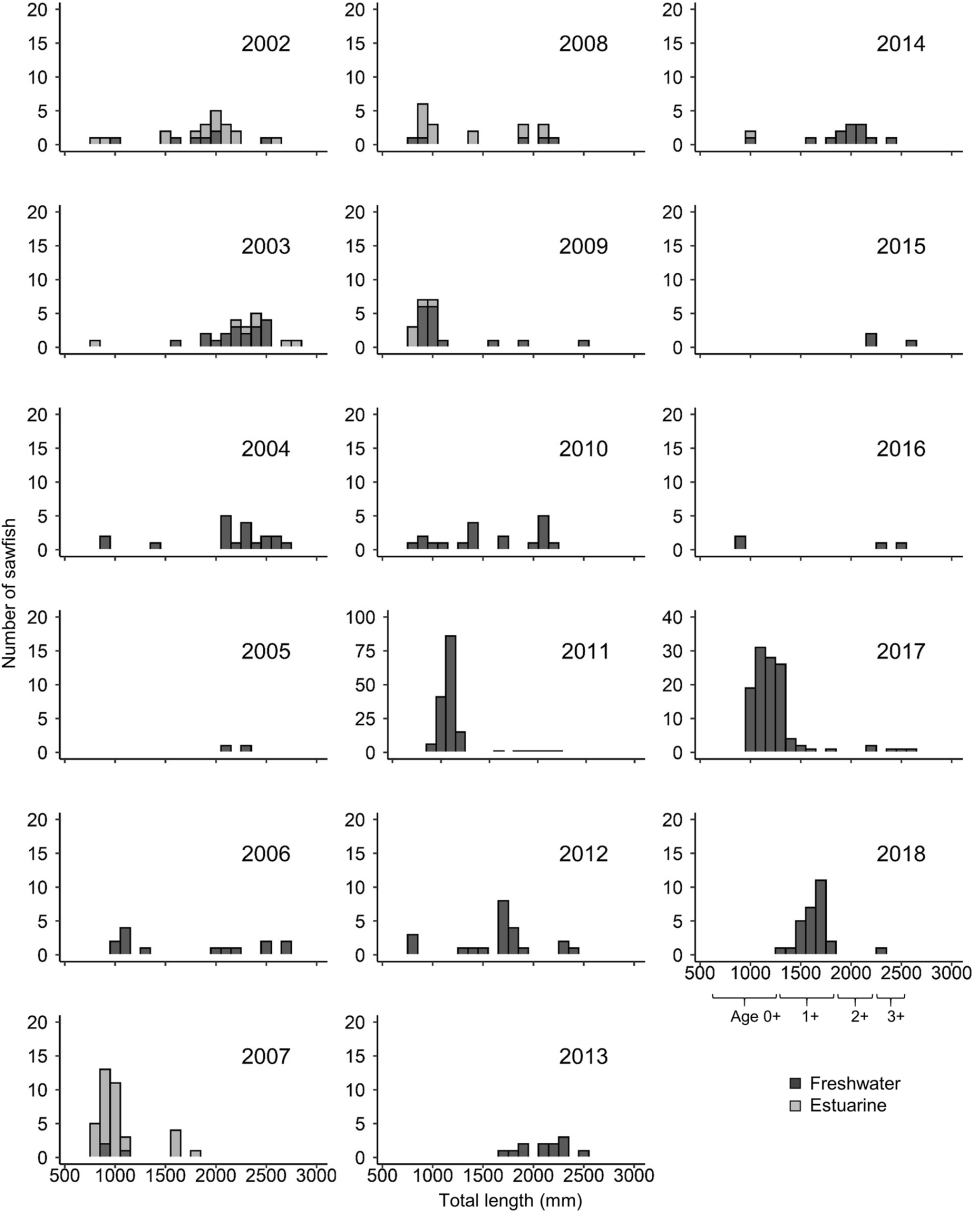
Annual size frequency distributions for freshwater sawfish. Data include all freshwater sawfish caught in estuarine and freshwater pools in the Fitzroy River in each year between 2002 and 2018, including all capture methods. Note that the y-axes for 2011 and 2017 are to a different scale than the other years because of higher catch rates. Approximate sizes for age 0+ through 3+ sawfish are noted under the x-axis. Years with high flow wet seasons (notably 2009, 2011, and 2017) are characterized by high frequency of young of year individuals (<1300 mm TL). Size classes in subsequent years are dominated by individuals recruited during these years, so that the majority of sawfish caught in the Fitzroy River can be traced back to a year with a high flow wet season and resulting high recruitment rates.

Including all size classes and capture methods, over 500 distinct individual freshwater sawfish were caught in the Fitzroy River between 2002 and 2018 in over 5,600 20-m net hours sampled. Individuals were caught up to 8 times over a period of 0–5 years, resulting in a total catch of 932 freshwater sawfish from 2002–2018. Freshwater pools were sampled in the early dry season in every year from 2003–2018 (mean ± SD annual survey effort 169 ± 132 20-m net hours), and in the late dry season in each year from 2002–2018, excluding 2005 (mean 113 ± 102 20-m net hours). Estuarine survey efforts were less consistent, with early dry season surveys conducted in 11 years between 2003 and 2016 (mean 59 ± 86 20-m net hours), and late dry season surveys conducted in 10 years between 2002 and 2015 (mean 45 ± 48 20-m net hours). Annual CPUE for all freshwater sawfish ranged from 0.008–0.45 sawfish 20-m net^−1^ h^−1^ in freshwater environments, and 0–0.28 sawfish 20-m net^−1^ h^−1^ in estuarine environments. Sawfish ranged in size from 763 to 2770 mm, with a female:male ratio of 1:0.96. In years with large wet season floods (notably 2009, 2011, and 2017), 0+ individuals were the dominant size class (TL < 1300 mm). Recruits from these years comprised the dominant size class in the river in subsequent years, so that 1+ individuals (between 1300–1600 mm TL) were the dominant size class in the immediate year after each high volume wet season, and large individuals (>2000 mm TL) dominated in the subsequent few years (Fig. 2). This was particularly evident from the high recruitment years of 2011 and 2017 (Fig. 2).

**Figure 3:**
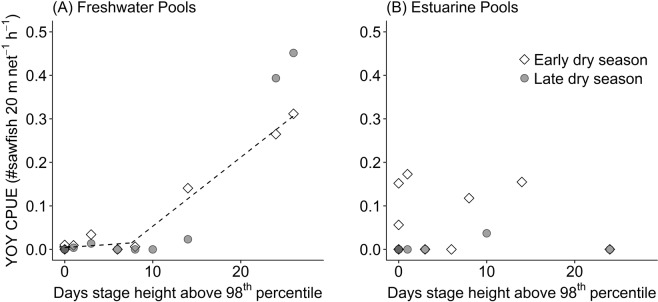
Relationship between recruitment of freshwater sawfish and wet season river flow. Graphs depict the relationship between the number of days during the wet season where river stage height exceeded the 98^th^ percentile of water levels on record (the best fit segmented model) and recruitment rates of freshwater sawfish in the Fitzroy River, measured as catch per unit effort (CPUE) of young of year (YOY) individuals (total length <1300 mm). The two highest freshwater CPUE values are from 2011 and 2017, the years with highest wet season volumes. In both (**A**) freshwater pools and (**B**) estuarine pools, CPUE predominately decreased between the early dry season (empty points) and late dry season (filled points). The segmented regression line for the best fit model is shown for the relationship between early dry season CPUE and stage height in freshwater pools.

In excess of 340 individual YOY freshwater sawfish were captured between 2002 and 2018. Young of year CPUE estimates tended to be highest in freshwater pools in the early dry season, ranging from 0–0.31 YOY sawfish 20-m net^−1^ h^−1^ annually, with the highest CPUE obtained in 2017. Within a year, CPUE sometimes varied substantially between nets, with per net CPUEs recorded as high as 6 YOY sawfish 20-m net^−1^ h^−1^. Young of year CPUE estimates were lower in the estuarine pools, ranging from 0–0.17 sawfish 20-m net^−1^ h^−1^ in the early dry season (Fig. 3). Catch per unit effort decreased from the early to late dry season in most years in both freshwater and estuarine environments, with low or no catches of YOY sawfish in freshwater pools in the late dry season, and no catches of YOY sawfish in estuarine pools in the late dry season except in 2002, where two sawfish were caught in a single net (CPUE = 0.037 sawfish 20-m net^−1^ h^−1^) (Fig. 3). The exceptions to this trend were catches from freshwater reaches in 2011 and 2017 (the two years with highest wet season volumes), where CPUE increased from the early to late dry season.

**Figure 4:**
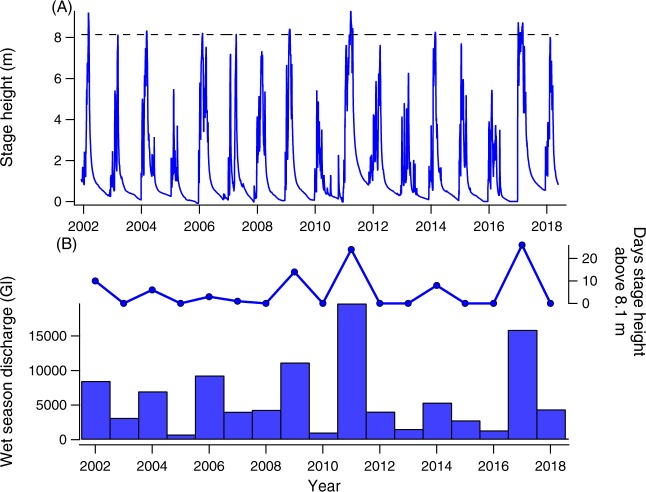
Inter- and intra-annual variation in Fitzroy River seasonal flow. (**A**) Cease-to-flow stage height of the Fitzroy River (measured at Willare) throughout the study period, showing the predictable intra-annual variation in river height associated with the wet and dry seasons. The dashed horizontal line indicates the stage height level that showed the most predictive power in freshwater sawfish recruitment models (98^th^ percentile of stage heights on record; see Table 2), indicating that sawfish predominantly use the brief and sporadic periods where stage height exceeds this level for recruitment to freshwater environments. (**B**) Inter-annual variation in total wet season discharge and the number of days stage height remained above 8.1 m (98^th^ percentile) in the Fitzroy River during the study period.

Wet season volume and duration for the Fitzroy River also showed substantial inter-annual variation between 2002 and 2018 (Fig. 4). Total wet season discharge volume ranged from a low of 758 Gl in 2005, to a high of 19,709 Gl in 2011, with a mean ± SD annual wet season discharge of 6,145 ± 5,334 Gl. When compared with CPUE estimates, a clear pattern emerged between the volume of the wet season and YOY recruitment to freshwater pools, with substantially more YOY sawfish caught in years with high volume wet seasons compared to years with low volume wet seasons (Fig. 3). Conversely, no obvious pattern was present in comparisons of YOY CPUE and wet season volume in estuarine pools in the early dry season or during the late dry season (Fig. 3). The relationship between freshwater recruitment and wet season volume was fit best by a segmented linear regression, which showed the lowest AICc, highest log likelihood, and highest R^2^ of any model (Table 1). When applied to all wet season descriptor variables, the number of days that river stage height remained above 8.1 m (equivalent to a discharge rate of above approximately 2,000 m^3 ^s^−1^), or the 98^th^ percentile of recorded river stage heights, emerged as the best predictor of recruitment (Table 2). The segmented regression set a breakpoint of 8 days where stage height exceeded 8.1 m. In years below this breakpoint, there was no or negligible recruitment of sawfish to freshwater pools, and in years above this breakpoint, we observed a steep increase in recruitment. While the regression set a breakpoint at 8 days, it should be noted that no appreciable recruitment to freshwater environments was observed at this level, similar to the breakpoints set in all other models, as this breakpoint is where the second portion of the segmented regression is rooted (see Fig. 3). Appreciable recruitment was only observed in years with 14 or more days where the river exceeded 8.1 m stage height (Table 2).

**Table 1:** Model selection table for models describing the relationship between freshwater sawfish recruitment and wet season discharge, with the best fit model chosen by the corrected Aikake’s Information Criterion (AICc), log likelihood and R^2^.

Model	AICc	ΔAICc	Log liklihood	R^2^
Segmented linear	−58.62	—	34.31	0.89
Exponential	−55.97	2.65	30.99	0.81
Power (aX^b^)	−55.82	2.80	30.91	—
Linear	−51.23	7.39	28.62	0.81

**Table 2:** Model selection table for models using different parameters describing the quality of the wet season (reported for Willare) to describe annual recruitment of freshwater sawfish, all fit with segmented linear regressions.

Wet season predictor	AICc	ΔAICc	Log likelihood	R^2^	Regression breakpoint ± S.D.	Min value with observed recruitment
Days stage height >8.1 m (98%)	−87.94	—	48.97	0.98	8 ± 1 days	14 days
Days stage height >7.3 m (95%)	−67.20	20.74	38.6	0.94	10 ± 4 days	17 days
Days stage height >5.6 m (90%)	−59.33	28.61	34.67	0.90	33 ± 5 days	45 days
Total discharge	−58.62	29.32	34.31	0.89	6602 ± 1472 Gl	9304 Gl
Early wet discharge (Dec–Jan)	−46.66	41.28	25.33	0.67	2219 ± 2921 Gl	1912 Gl
Maximum stage height	−44.02	43.92	27.01	0.72	8.1 ± 0.1 m	8.2 m
Mid wet discharge (Feb–Mar)	−43.91	44.03	26.96	0.73	4469 ± 1647 Gl	6317 Gl
Days stage height >2.2 m (75%)	−41.19	46.75	25.59	0.67	108 ± 11 days	93 days
Days stage height >0.9 (50%)	−37.83	50.11	23.92	0.60	174 ± 40 days	141 days
Late wet discharge (Apr-May)	−26.50	61.44	18.25	0.19	818 ± 1655 Gl	139 Gl

The breakpoints of these regressions are listed, but note that recruitment was still negligible at these breakpoints for all parameters as the second segment of the regression is anchored through this point (see Fig. 3). Therefore, the minimum value at which recruitment was observed is also noted for each variable. Numbers in parentheses for listed stage heights indicate the percentile of the stage height within all stage heights reported for Willare between 1998 and 2018.

## Discussion

Long-lived and slow-maturing species including sawfishes are prone to extinction and require long-term ecological investigations in order to reveal patterns in life history and population dynamics^17,23^, particularly when ecosystem drivers, such as dryland flood rhythms, show high variability^13,14^. The current 17 year study provides an unprecedented perspective from which to clarify the hydrological requirements of the Fitzroy River catchment for rearing freshwater sawfish. Specifically, we report a clear relationship between wet season volume and recruitment success of freshwater sawfish in the Fitzroy River, highlighting the essential importance of brief and infrequent high water level periods for recruitment. Quantifying this recruitment threshold will be exceptionally valuable for informing management of water resources in the Fitzroy River, and highlights the benefit of long-term ecological monitoring for underpinning ecologically sustainable water resource and catchment management.

### Recruitment and flood volumes

All freshwater sawfish captured in the freshwater and estuarine reaches of the Fitzroy River during the study were juveniles, reaching a maximum of 2.6 m. While fully mature freshwater sawfish including pregnant females have been observed in freshwater environments in other systems such as Lake Nicaragua^35,36^, only immature individuals have ever been observed in the freshwater reaches of rivers in Australia^20–23,25^. This could be due to the confined space in dry season pools in these systems, which would restrict movement of larger mature individuals, or different life histories observed in the two genetically distinct sub-populations^18^, which also show slightly different morphologies including rostral tooth counts^21,37^. The absence of mature sawfish in the Fitzroy River emphasizes the importance of this system specifically as a nursery habitat.

The best wet season predictor of freshwater sawfish recruitment rates was time spent above the 98^th^ percentile of stage height (8.1 m at Willare), with appreciable recruitment into freshwater environments only observed in years with at least 14 days where river stage height was above this level. This indicates that freshwater sawfish most strongly rely on the brief periods of highest water levels for recruitment, rather than sustained medium to low water level periods, and that there is a high threshold in water levels and wet season discharge that allows freshwater sawfish to enter and survive in freshwater environments. This stage height threshold is not trivial, having not been reached at all in more than 50% of years during this study, and the threshold of 14 days where river height exceeded 8.1 m occurred in only 3 years (~18%) of the study period, resulting in one year with moderate recruitment (2009), and two years with high rates of recruitment (2011 and 2017).

There are several factors that could contribute to these observed patterns in recruitment. First, while there is almost always sufficient water to connect the lower reaches of the Fitzroy River at some point during the wet season, YOY freshwater sawfish may require extended periods of high water levels or a higher threshold water level to traverse through the estuarine pools into freshwater environments. High water levels may also make it more energetically efficient for sawfish to migrate because of the presence of low velocity areas around eddies or particularly on the floodplain, whereas low water levels would constrain sawfish to travelling in main channel shallow riffle zones that typically have high water velocities^38,39^, and could also concentrate predation pressure. When these high water levels are not met, sawfish may become trapped in the estuary^32^. This is supported by the lack of a relationship between estuarine YOY CPUE and wet season volume, suggesting that freshwater sawfish are pupped and may reach macrotidal estuarine environments regardless of the magnitude of flooding. However, survival of YOY sawfish in the estuary appears to be low, with YOY CPUE decreasing to zero or near zero by the late dry season in all years of the study. Estuarine pools, as well as nearby freshwater pools that sawfish may be able to access at low water levels, host a higher density of predators than freshwater environments, including estuarine crocodiles (*Crocodylus porosus*), and bull sharks (*Carcharhinus leucas*)^40^. These pools also host several competitor species, such as dwarf sawfish (*Pristis clavata*), which enter estuarine environments in the dry season^20,23^. Whether due to elevated predation, competition, or both, the low estuarine CPUE in the late dry season indicates that in years where fish are not able to migrate to upstream freshwater environments, overall recruitment of freshwater sawfish to the Fitzroy River nursery is likely negligible.

Similar to estuarine pools, CPUE in freshwater pools also decreased during the dry season in most years, though these drops in CPUE were proportionally less than those observed in the estuary. The decrease in freshwater CPUE is most likely due again to predation by estuarine crocodiles and bull sharks, as well as freshwater crocodiles (*Crocodylus johnstoni*) and humans^40,41^. Sawfish in freshwater pools may also succumb to harsh environmental conditions including high temperatures and low dissolved oxygen levels^31,42^. Conversely, in the two years with the highest wet season volumes during the study period, 2011 and 2017, CPUE increased between the early and late dry season. Sawfish may have higher survival rates in wetter years because of increased dry season habitat quality and river productivity due to the high water levels and a large flood pulse^28,43^. It is also possible that the sustained high water levels during the wet season and early dry season in such years allows for a prolonged recruitment period due to extended pool connectivity. In other years, the pools become isolated once river flow ceases, meaning that sawfish are unable to move between different pools within a dry season. This also means that the observed drops in CPUE in low flood years are not due to sawfish relocating to unsampled locations. It is possible that these drops in CPUE in low flood years could be due to changes in sawfish behaviour between the early and late dry season, for example in reaction to higher water temperatures and low food resources in the late dry season. However, previous tracking studies have shown that sawfish reliably move between deep refuge habitats and shallow foraging areas for the duration of the dry season^31^, and that sawfish are more active under higher temperature regimes in the late dry season compared to the early dry season^42^. Neither of these trends would reduce catchability of sawfish, and therefore behavioural differences within a dry season are unlikely to account for the divergent patterns in CPUE between high and low flood years.

It is also possible that a greater freshwater pulse into King Sound and the surrounding environment increases the number of recruits entering the Fitzroy River nursery. Relatively little is known about the reproductive process or the triggers for parturition in freshwater sawfish, other than the speculated wet season period for pupping^20,21,32^. Genetic data indicate that females may be philopatric^44,45^, suggesting that a larger freshwater pulse would not necessarily draw more females from long distances, and a gestation period of approximately five months^35^ would generally preclude a large freshwater pulse spurring higher reproductive rates for individual females within the same season. However, a large freshwater pulse or an extended freshwater pulse may help to direct females more specifically to pup at the mouth of the Fitzroy River, or help to guide newly born pups upriver throughout the wet season parturition period, introducing higher numbers of recruits into the river system in these years. Prolonged high freshwater flows may also allow newly born pups to migrate upriver immediately after birth, whereas less reliable flow periods may force pups to remain longer in the precarious macrotidal estuarine environments subject to high predation risk before they are able to begin their migration.

Additionally, it is possible that recruitment to the Fitzroy River nursery is occurring in poor wet season years outside of our survey areas, such as the freshwater pools just beyond the tidal limit. However, the connectivity of the Fitzroy River during the wet season allows for a redistribution of sawfish throughout all regions of the river. As a result, regardless of where new recruits reside in their first year, they may redistribute and be found in other regions in subsequent years. For example, in 2017 more than 100 YOY freshwater sawfish were tagged in Camballin Pool (river km 150), but in the following year, only two of these tagged fish were recaptured in the same pool, along with 21 new individuals, indicating a redistribution of fish from unsampled to sampled locations during the 2017–2018 wet season. The annual size frequency distributions for freshwater sawfish in the Fitzroy River show that the majority of fish caught can be attributed to a recruitment class for a year with a high sampled YOY CPUE (Fig. 4), indicating that our freshwater survey locations provided a reasonably representative sample of the sawfish present in other freshwater regions of the river.

Regardless of the specific breakdown of factors contributing to the observed patterns in recruitment, it is clear that wet season volume distinctly impacts the ability of freshwater sawfish to recruit into the Fitzroy River nursery. This relationship creates what we describe as an interannual boom-or-bust cycle for freshwater sawfish, where annually there is either negligible recruitment or high recruitment depending on the quality of the prior wet season. As a result, this species relies almost entirely on infrequent years with substantial wet season floods to replenish populations in the Fitzroy River. Such boom-or-bust cycles relating to flood patterns are fairly common for aquatic species in dryland river systems^9,10,29^ as well as terrestrial species in arid environments^15,46,47^. For example, the volume of freshwater flows is linked to year class strength of catadromous barramundi (*Lates calcarifer*), with recruits surviving better in wetter years because of increased accessibility to and carrying capacity of freshwater nursery habitats^28^. However, this type of pattern in recruitment highlights the delicate balance of environmental conditions that must be maintained in order to ensure the future recruitment and persistence of freshwater sawfish in the Fitzroy River, and also emphasizes the necessity of long-term ecological data for understanding these ecosystems^16,17^.

### Conservation implications

The boom-bust recruitment pattern and high wet season stage height threshold for recruitment emphasize the importance of maintaining the periods of highest flows during the wet season period in the Fitzroy River, which would preclude the building of major dams or weirs, or any substantial water diversion. By design, these structures seek to diminish the most extreme natural variance in river flow in order to provide more stable hydrologic conditions for agricultural purposes, resulting in diminished flow during the wet season and potentially increased flow during the dry season. In the case of freshwater sawfish, successful recruitment into their riverine nursery relies not only on the years with high volume wet seasons, but specifically the relatively brief periods of extreme highest river stage height within these wet seasons. Considering that the high water level conditions enabling recruitment are not met in the majority of years, decreasing the existing rate and volume of wet season flooding could have substantial impacts on the ability of freshwater sawfish to recruit into their nursery. At its most extreme, dampening wet season flow and decreasing discharge could eliminate the extreme high water level periods in the Fitzroy River that freshwater sawfish rely on, reducing overall recruitment to negligible levels. This process may at least partially account for the low numbers of freshwater sawfish found in the Ord River in the east Kimberley, which falls under a similar climate to the Fitzroy River and historically boasted similar flow regimes and habitats, but now hosts multiple large dams that have significantly disrupted river flow dynamics^48,49^. While freshwater sawfish are present in the Ord River^20^, the population is barely detectable^49^. In addition to altering flow regimes, dams and other barriers such as weirs or vehicle crossings impede fish migration, particularly when river levels drop in the dry season. The low survival of freshwater sawfish in estuarine pools in the Fitzroy River emphasizes the importance of preserving unimpeded passage of sawfish from the estuary into freshwater environments.

The Fitzroy River is arguably the most important identified nursery for freshwater sawfish globally, though it is possible that there are other proficient nursery grounds for this species in areas that have not been thoroughly studied. For example, there is evidence that rivers in Brazil and Nicaragua may also hold important pupping grounds^18^. For the known nursery habitat in the Fitzroy River, we suggest further anthropogenic disturbance should be minimized to maintain what is still a relatively pristine habitat^20^, a recommendation that would also be important for any other nurseries in other subpopulations, as this study has demonstrated the importance of natural flow regimes and habitats for this species. The Fitzroy River is a dynamic system characterized by a delicate balance of extreme conditions. Our results show that during the wet season freshwater sawfish rely on the utmost of these extremes to recruit into the system and replenish their population, a process that is already limited to relatively few years. Even slight deviations from the natural flow regimes in such a dynamic environment may substantially affect the success of freshwater sawfish populations in this ecosystem. Ultimately, successful management of the Fitzroy River for conservation of freshwater sawfish and other aquatic species will entail policies that seek to both preserve the most extreme wet season flow regimes and maintain suitable habitat quality for these fish throughout the dry season.
